# Supplementary material for: Catalyst-free selective oxidation of C(sp3)-H bonds in toluene on water
Source: Nat Commun. 2024 Jul 20;15:6127. doi: 10.1038/s41467-024-50352-7 (PMC11271591; doi:10.1038/s41467-024-50352-7)
Supplement: Supplementary file 1 — Supplementary Information [file 41467_2024_50352_MOESM1_ESM.pdf]

Supplementary Information

Catalyst-free selective oxidation of C(sp^3)-H bonds in toluene on water

Kyoungmun Lee[1], Yumi Cho[2], Jin Chul Kim[2], Chiyoung Choi[3], Jiwon Kim[1], Jae Kyoo Lee[3,4], Sheng Li[1,5], Sang Kyu Kwak[6,*] & Siyoung Q. Choi[1,5,*]

[1]Department of Chemical and Biomolecular Engineering, Korea Advanced Institute of Science and Technology (KAIST), Daejeon 34141, Republic of Korea

[2]School of Energy and Chemical Engineering, Ulsan National Institute of Science and Technology (UNIST), UNIST-gil 50, Ulju-gun, Ulsan 44919, Republic of Korea

[3]Department of Applied Bioengineering, Graduate School of Convergence Science and Technology, Seoul National University, Seoul 08826, Republic of Korea

[4]Research Institute for Convergence Science, Seoul National University, Seoul 08826, Republic of Korea

[5]KAIST Institute for the Nanocentury, KAIST, Daejeon 34141, Republic of Korea.

[6]Department of Chemical and Biological Engineering, Korea University, Seoul 02841, Republic of Korea

*E-mail: skkwak@korea.ac.kr (Kwak, S. K.); sqchoi@kaist.ac.kr (Choi, S. Q.)

Table of Contents

Supplementary Figures

Supplementary Fig. 1: Size distribution of the generated emulsion droplets 1
Supplementary Fig. 2: UV-visible absorption intensity corresponding to the H_2O_2 concentration 2
Supplementary Fig. 3: Concentration of H_2O_2 generated by different oil-water interfaces 3
Supplementary Fig. 4: Additive- and initiator-free radical polymerization on water............................ 4
Supplementary Fig. 5: Size distribution of the created emulsion droplets with toluene 5
Supplementary Fig. 6: Effect of ultrasound on the on water reactions... 6
Supplementary Fig. 7: Effect of radical scavenger on toluene oxidation 7
Supplementary Fig. 8: Degradation of hexadecane and its effect on the on water reactions 8
Supplementary Fig. 9: GC-MS analysis of toluene oxidation on water 9
Supplementary Fig. 10: Microdroplet mass spectrometry analysis 10
Supplementary Fig. 11: Influence of dissolved oxygen on H_2O_2 generation 11
Supplementary Fig. 12: The reaction state configurations of toluene in bulk oil 12
Supplementary Fig. 13: The reaction state configurations of toluene at the oil-water interfaces......... 13
Supplementary Fig. 14: DFT optimized structure and molecular electrostatic potential (MEP) map.. 14
Supplementary Fig. 15: Configurations of the H-abstraction reaction states of benzaldehyde 15
Supplementary Fig. 16: Evaluation of H_2O_2 production at various oil-water interfaces 16
Supplementary Fig. 17: GC-MS analysis of benzene oxidation on water 17
Supplementary Fig. 18: GC-MS analysis of o-xylene oxidation on water 18
Supplementary Fig. 19: GC-MS analysis of m-xylene oxidation on water 19
Supplementary Fig. 20: GC-MS analysis of p-xylene oxidation on water 20
Supplementary Fig. 21: GC-MS analysis of 1,2,4-trimethylbenzene oxidation on water 21
Supplementary Fig. 22: Molecular dynamics system with toluene at the oil-water interface 22
Supplementary Fig. 23: Molecular dynamics system with benzaldehyde at the oil-water interface.... 23

Supplementary Tables

Supplementary Table 1: On water activity for selective toluene oxidation.. 24
Supplementary Table 2: Comparison of on water toluene oxidation with catalytic studies................. 25
Supplementary Table 3: Influence of dissolved oxygen concentration on toluene oxidation 26
Supplementary Table 4: Effect of the π-hydrogen bond strength on toluene oxidation....................... 27
Supplementary Table 5: Impact of chain length of hydrocarbon oils on toluene oxidation................. 28
Supplementary Table 6: Selective activation of $C(sp^3)$-H bonds in various aromatic compounds 29

Supplementary References 30

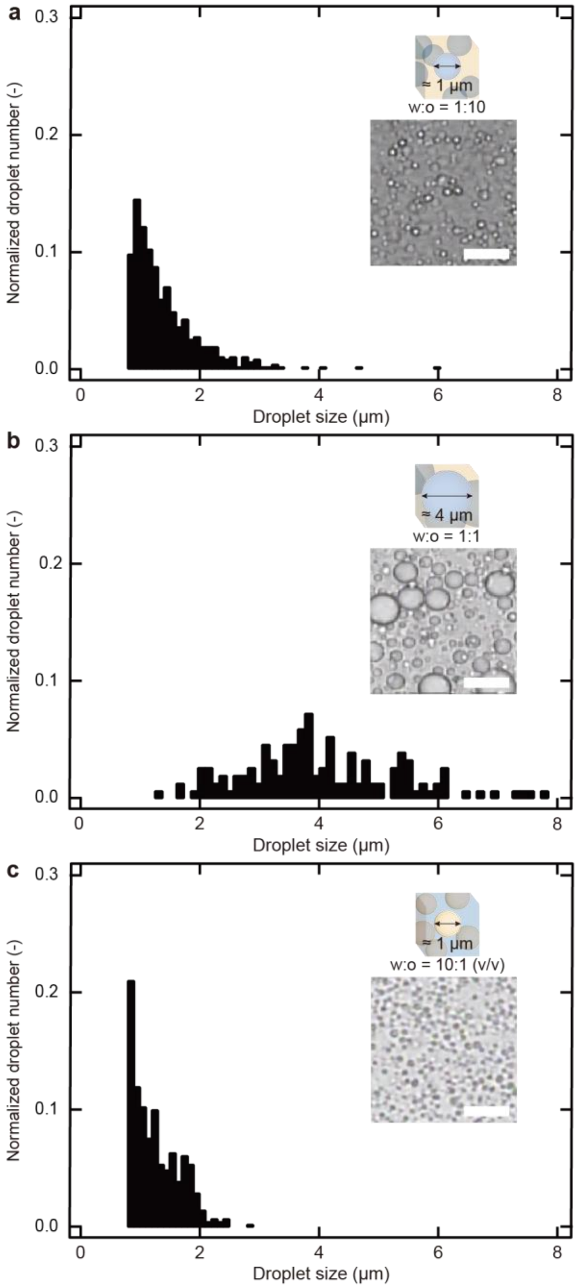

Supplementary Fig. 1. Size distribution of the generated emulsion droplets. Three different oil-water interfaces were created by emulsifying 1:10 (**a**), 1:1 (**b**), and 10:1 (**c**) (v/v) mixtures of water and hexadecane. The inset images depict the produced emulsion droplets at 30 min sonication time (scale bar: 10 µm).

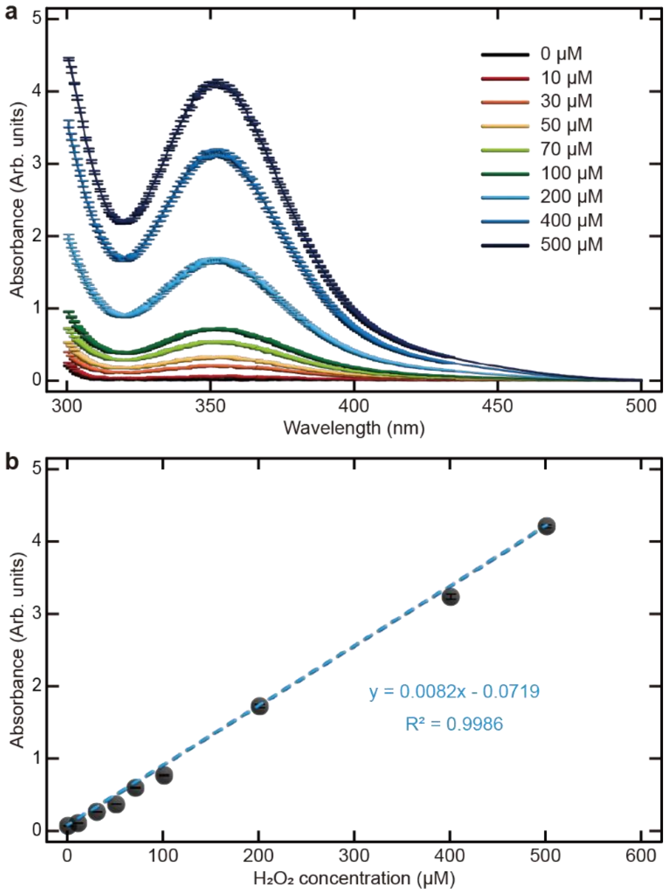

Supplementary Fig. 2. UV-visible absorption intensity corresponding to the H_2O_2 concentration. Absorption peak at 353 nm results from the oxidation of the I^- ion to the I_3^- ion by the catalytic activity of ammonium molybdite in the presence of H_2O_2. **a,** UV-visible spectra of aqueous solutions containing different concentrations of H_2O_2. **b,** Linear increase of absorption peak at 353 nm with increasing H_2O_2 concentration. Higher concentrations above 500 μM were measured after dilution.

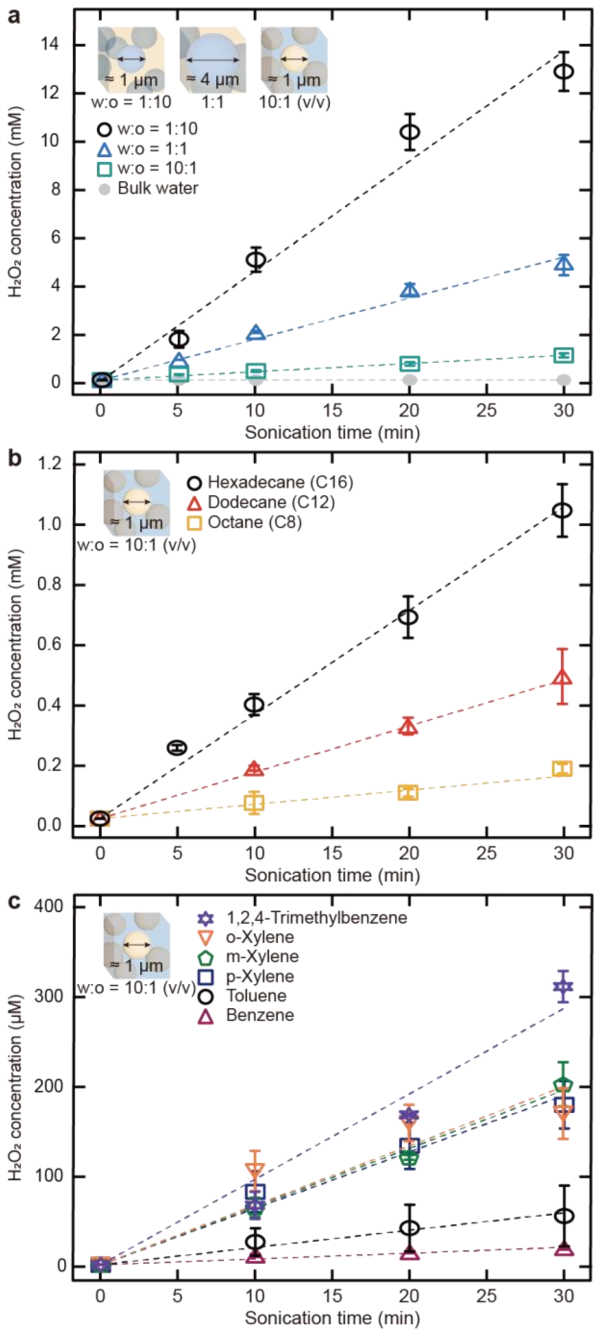

Supplementary Fig. 3. Concentration of H_2O_2 generated by different oil-water interfaces. a, Concentration of H_2O_2 generated by emulsifying 1:10, 1:1, and 10:1 (v/v) mixtures of water and hexadecane. In bulk water, the H_2O_2 concentration was below the detection threshold. **b, c,** The effect of chain length of hydrocarbon oils (**b**) and oil types (**c**) on the production of H_2O_2 in 10:1 (v/v) water and oil mixtures. The standard deviation of three measurements is denoted by each error bar.

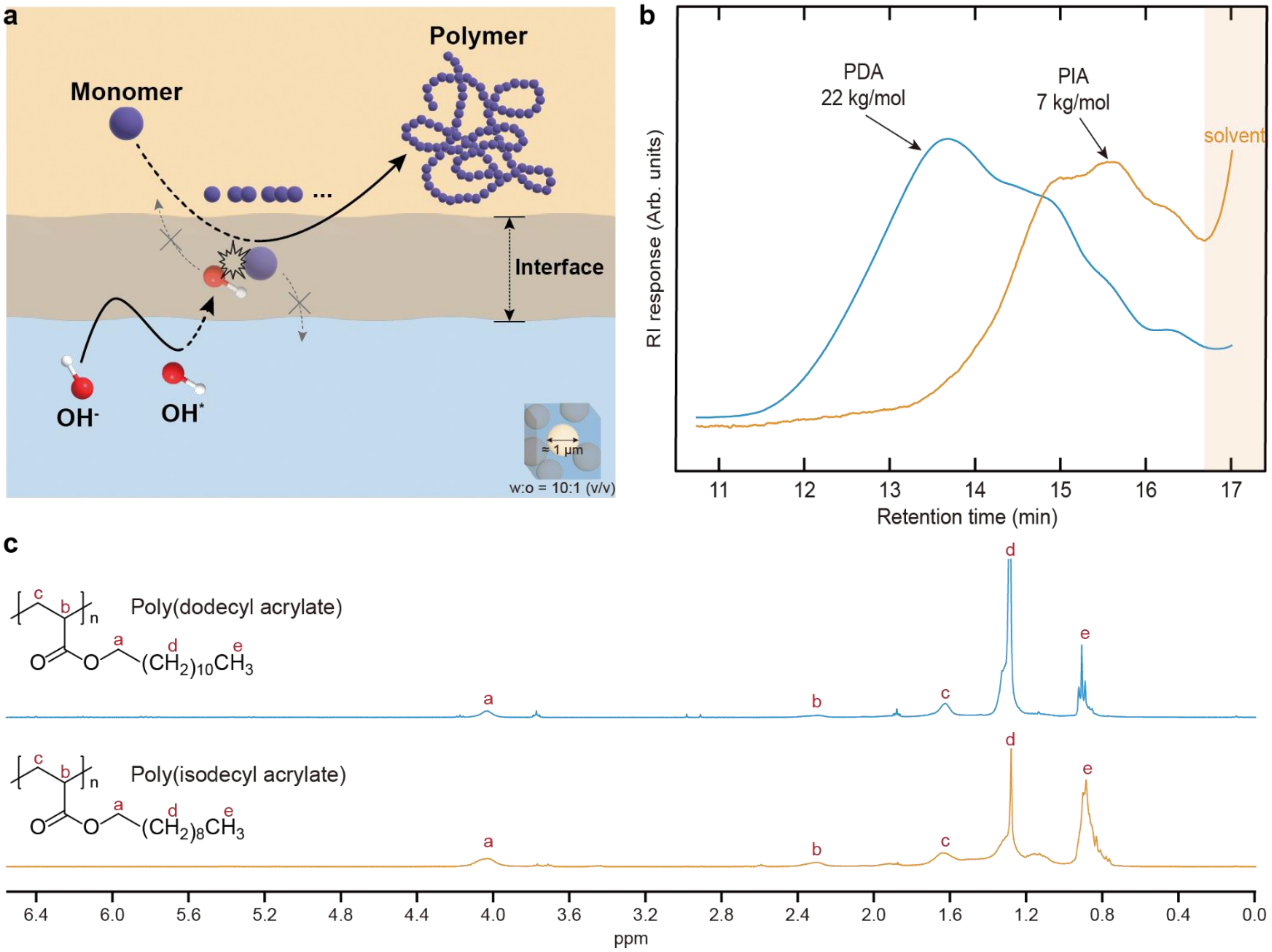

Supplementary Fig. 4. Additive- and initiator-free radical polymerization on water. a, Schematic illustration of the free radical polymerization of oil-soluble monomers initiated by the transport of OH· across the interfaces. **b,** Representative tetrahydrofuran SEC traces of the synthesized poly(dodecyl acrylate) (PDA) and poly(isodecyl acrylate) (PIA). **c,** ^{1}H NMR spectra (400 MHz, $CDCl_3$) of PDA and PIA synthesized by oil-water interfaces.

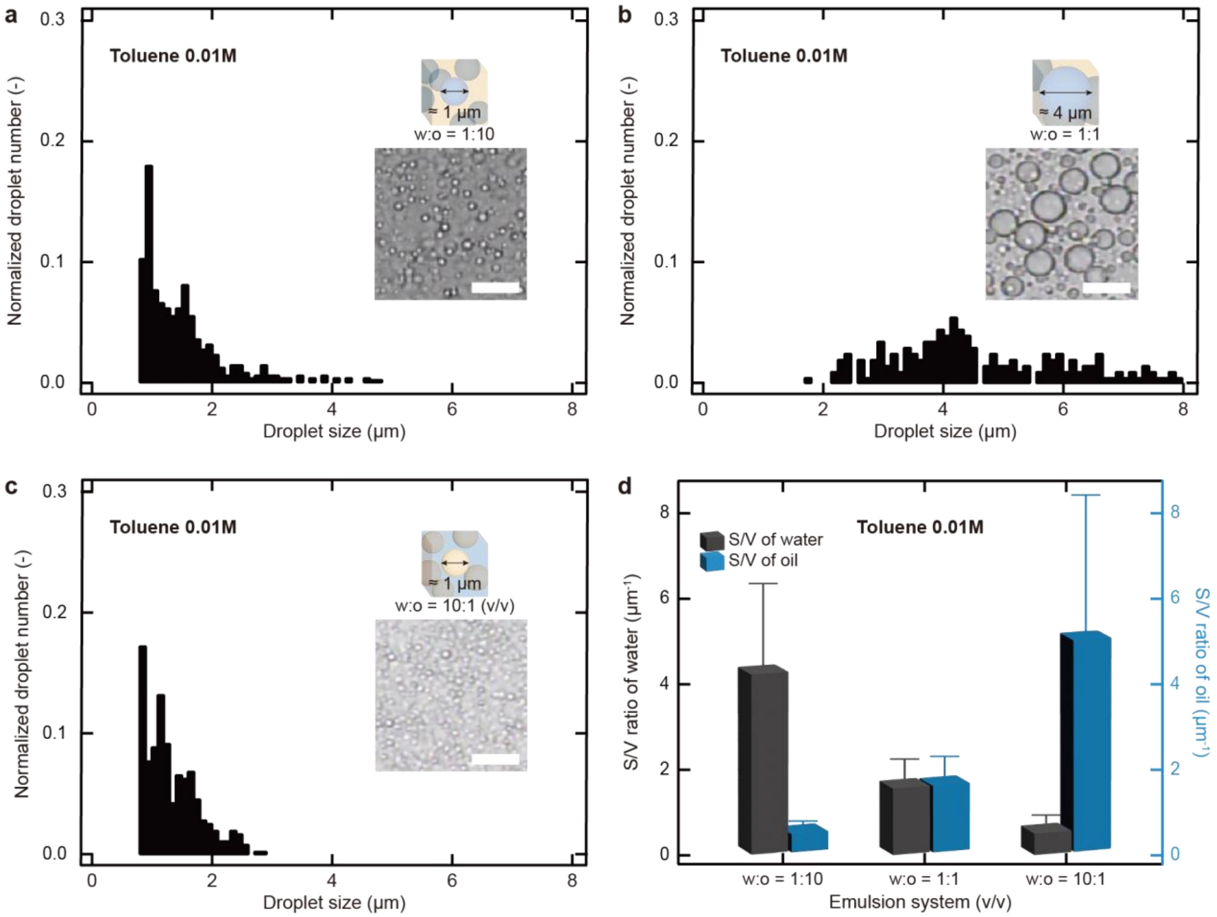

Supplementary Fig. 5. Size distribution of the created emulsion droplets with toluene. a-c, Three different oil-water interfaces were created by emulsifying 1:10 (**a**), 1:1 (**b**), and 10:1 (**c**) (v/v) mixtures of water and hexadecane solution (toluene 0.01 M). The inset images depict the produced emulsion droplets at 30 min sonication time (scale bar: 10 μm). **d,** S/V ratios of the water and oil phases (toluene 0.01 M) in three different emulsions. Three measurements are represented by each error bar, which stands for the standard deviation.

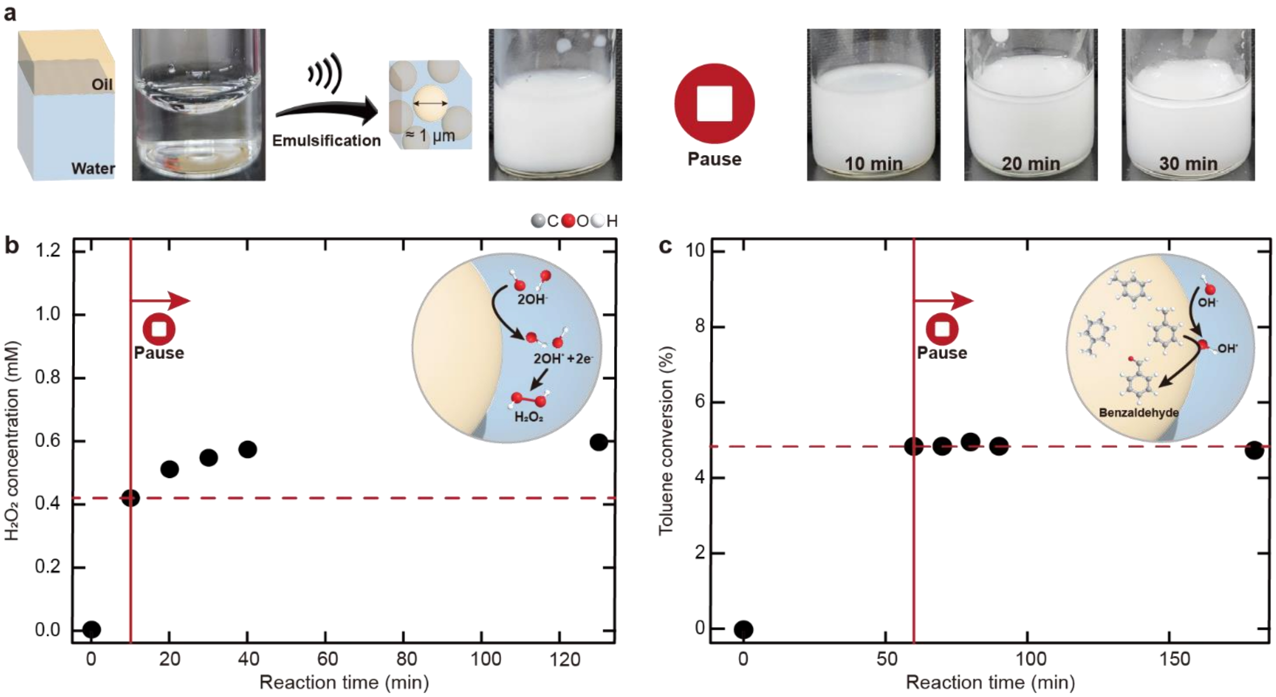

Supplementary Fig. 6. Effect of ultrasound on the on water reactions. a, Schematic diagram of the experimental procedure. Solutions of hexadecane and water in a 10:1 (v/v) ratio were subjected to ultrasonication for the desired time. Prompt reactions were observed subsequent to the end of ultrasonication. **b,** Concentration of H_2O_2 after 10 min exposure to ultrasound. Dotted line represents the H_2O_2 concentration at 10 min. **c,** Conversion of toluene after1 h exposure to ultrasound (0.01 M toluene). Dotted line represents the conversion of toluene at 1 h.

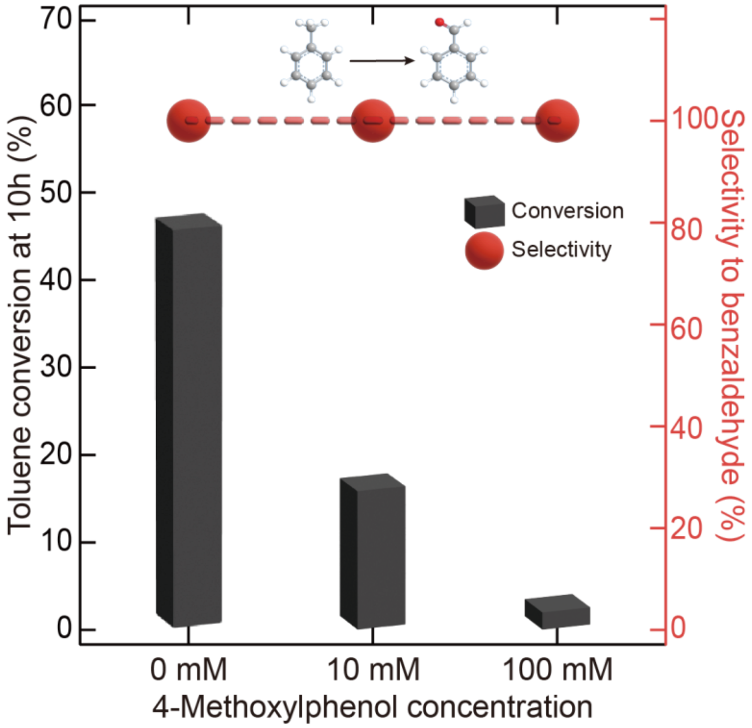

Supplementary Fig. 7. Effect of radical scavenger on toluene oxidation. Toluene conversion and benzaldehyde selectivity upon addition of varying concentrations of 4-methoxyphenol: $[Toluene]_0$ = 0.01 M, 1 atm oxygen, 25 °C (298 K), water to oil ratio 10:1 (v/v).

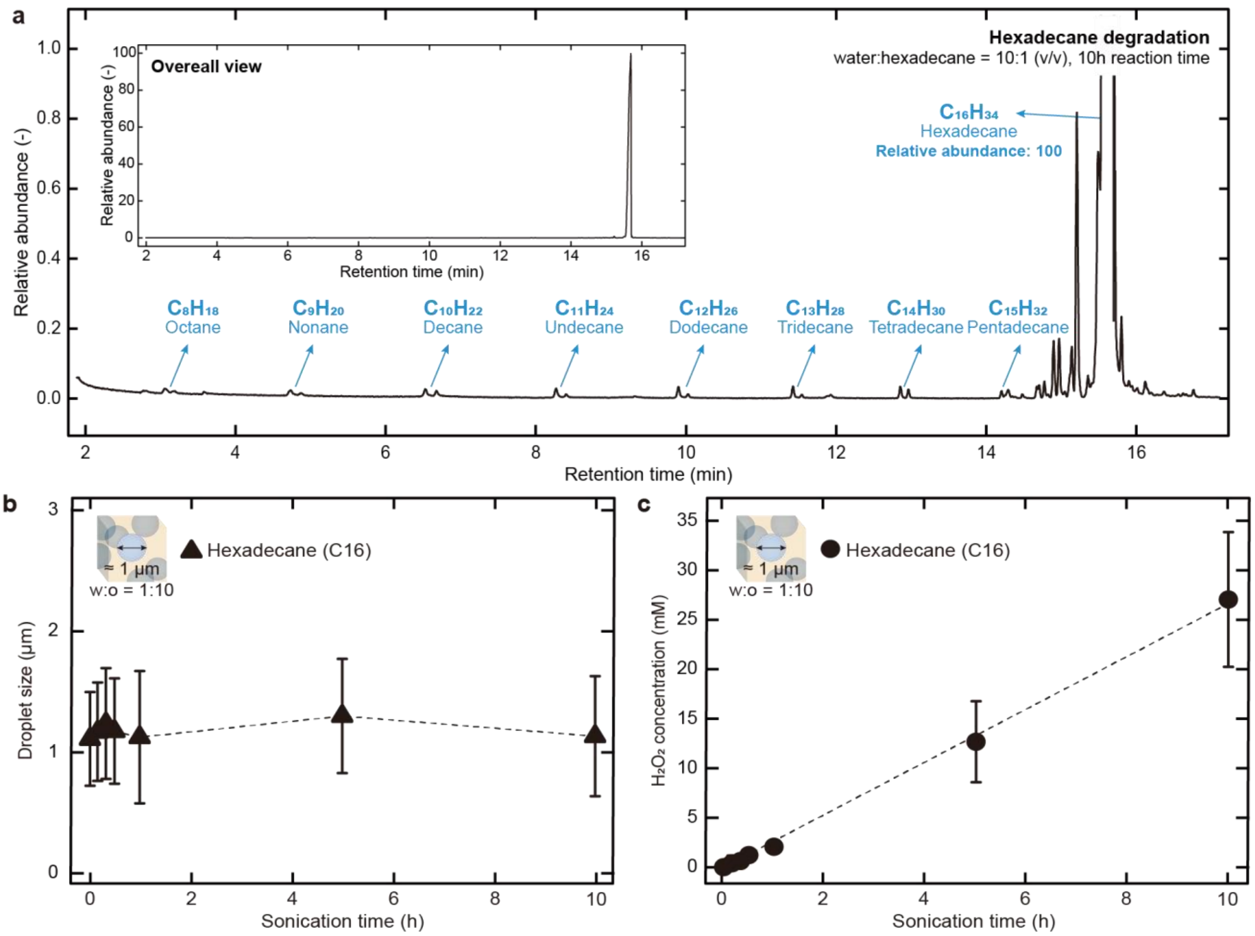

Supplementary Fig.8. Degradation of hexadecane and its effect on the on water reactions. a, GC-MS analysis of hexadecane degradation. The inset image depicts the overall view of elution profiles. Experimental conditions: 1 atm nitrogen, 25 ^{o}C (298 K), water to hexadecane ratio 10:1 (v/v), 10 h reactions. **b, c,** Diameter of oil droplets (**b**) and H_2O_2 concentration (**c**) generated by emulsifying 1:10 (v/v) mixtures of water and hexadecane. Each error bar denotes the standard deviation of three measurements.

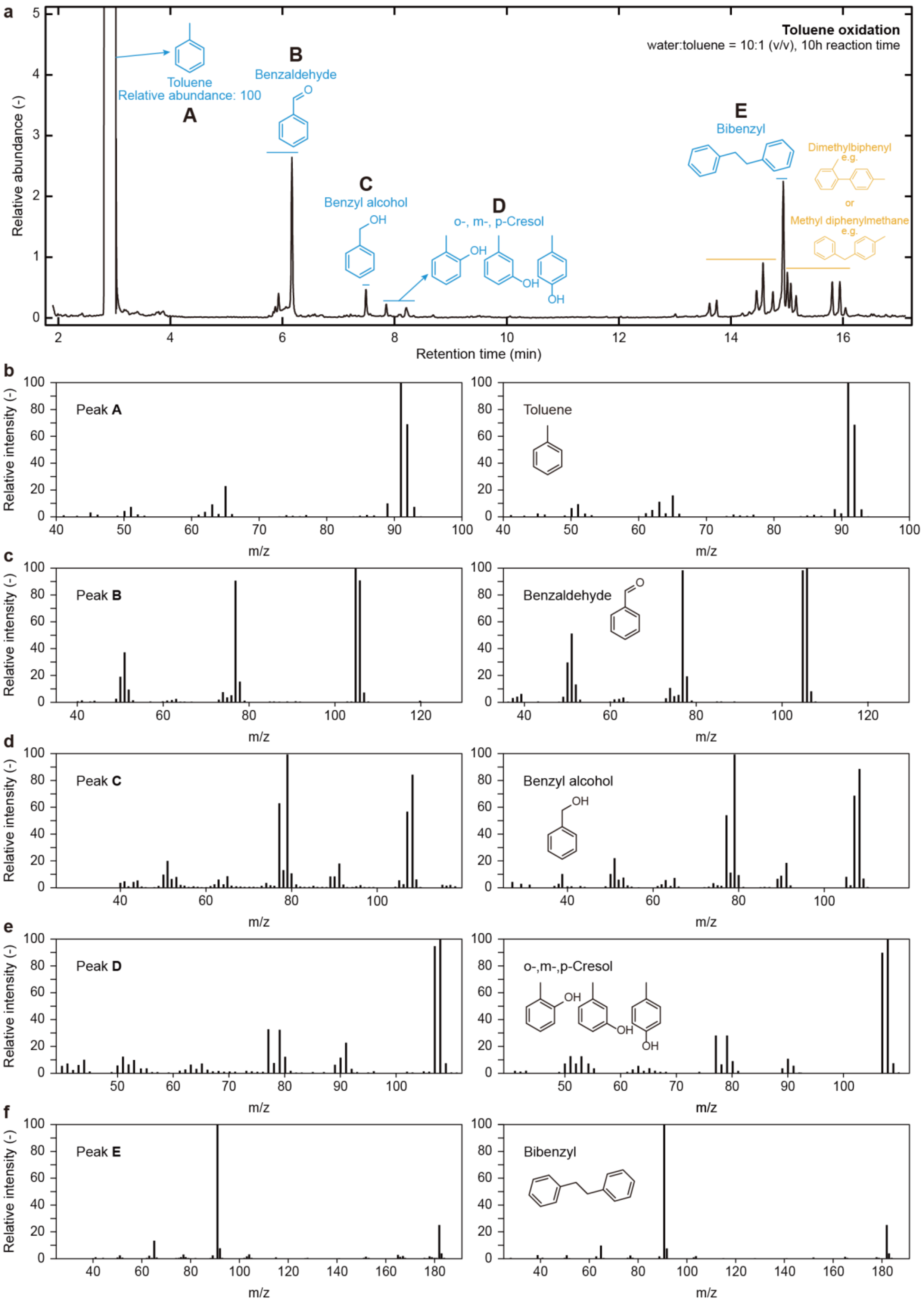

Supplementary Fig. 9. GC-MS analysis of toluene oxidation on water. a, Elution profiles of GC measurements. **b-f**, Mass spectra of the oxidized products: 1 atm oxygen, 25 ºC (298 K), 10 M toluene, water to toluene ratio 10:1 (v/v), 10 h reaction.

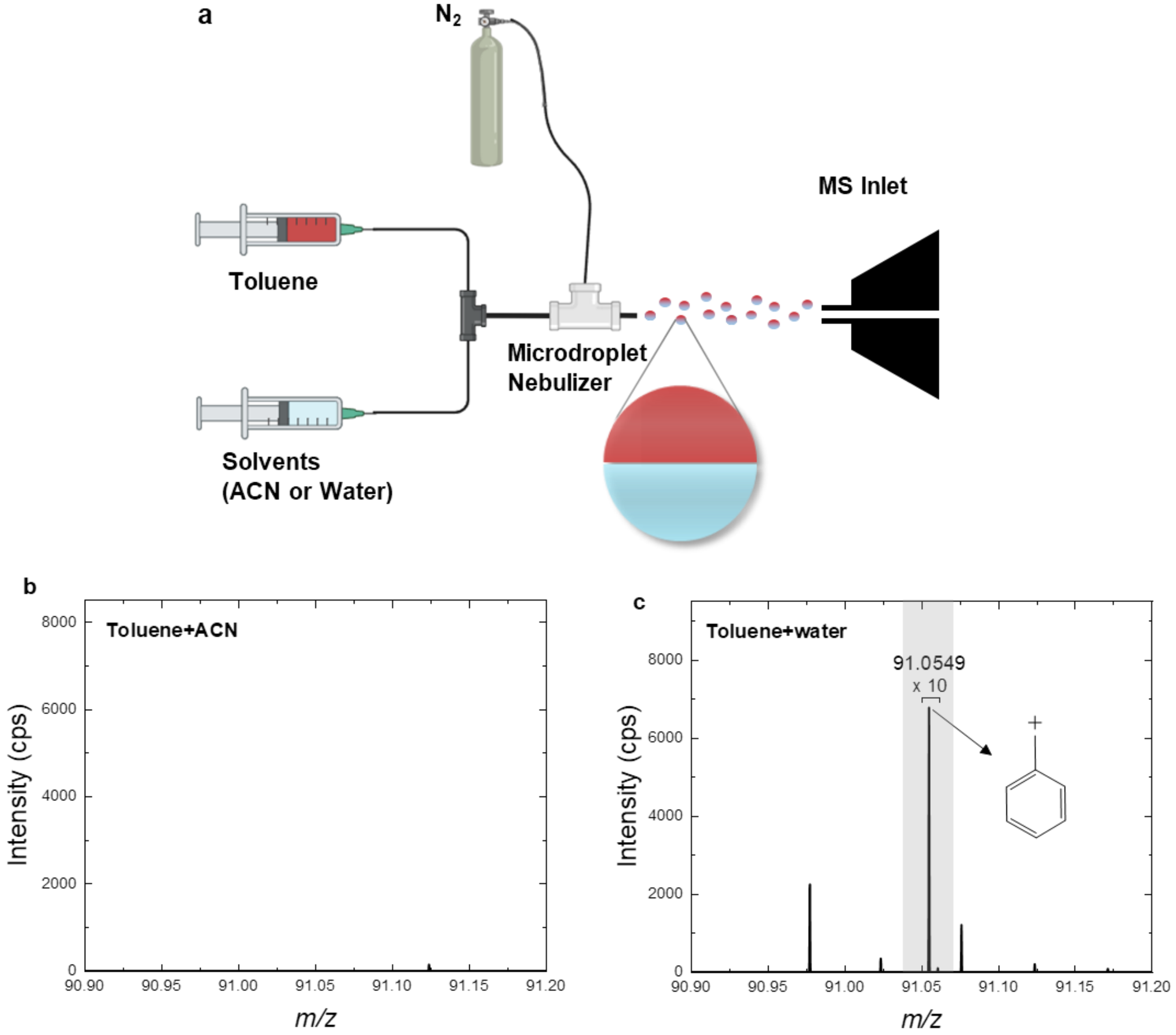

Supplementary Fig. 10. Microdroplet mass spectrometry analysis. a, Schematic diagram of the microdroplet spectrometry setup. Microdroplets, generated by nebulizing a mixture of toluene and either acetonitrile (ACN) or water with N_2 gas were directly injected into a mass spectrometer for the analysis of reaction intermediates. **b, c,** The mass spectra obtained using different solvents, ACN (**b**) and water (**c**). The MS peak at *m/z* 91.0529 corresponding to $C_7H_7^+$ was observed in microdroplets only with toluene and water interfaces, not toluene and ACN interfaces.

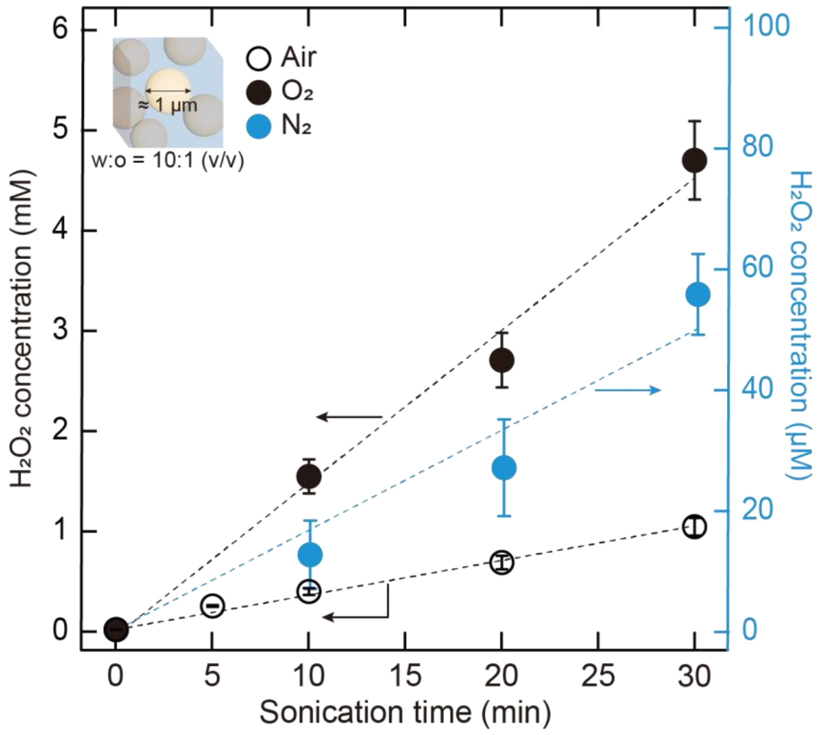

Supplementary Fig. 11. Influence of dissolved oxygen on H_2O_2 generation. Both water and oil were exposed to a 20-minute purging process with air or O_2 or N_2 gas prior to emulsification. Standard deviations of three measurements are indicated by each error bar.

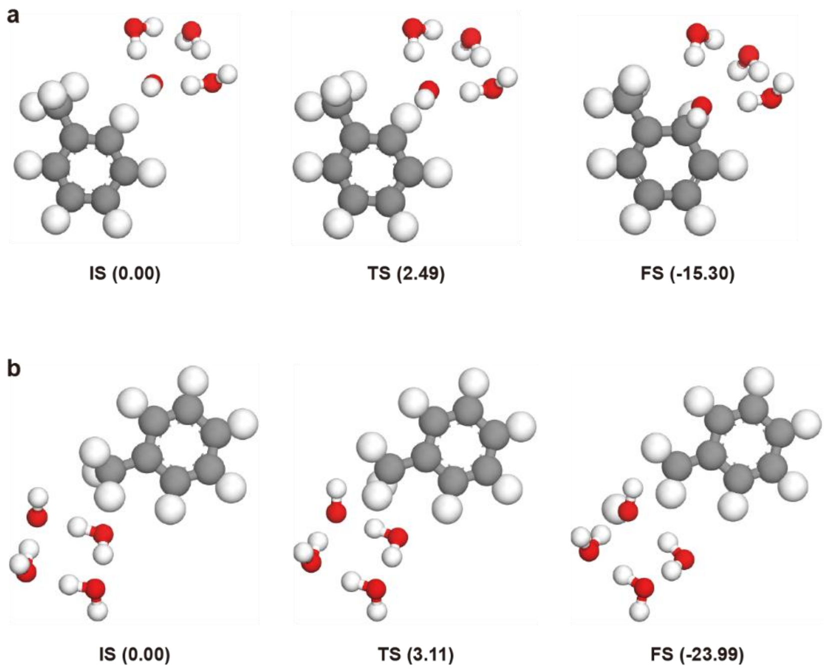

Supplementary Fig. 12. The reaction state configurations of toluene in bulk oil. a, OH-addition and **b,** H-abstraction. Toluene and water molecules are shown by ball-and stick model. In the color scheme, C, O, and H atoms are colored in gray, red, and white, respectively. The activation energy and heat of reaction are represented by the number in parenthesis below each snapshot in the unit of kcal/mol. IS, TS, and FS in each reaction mechanism represent initial state, transition state, and final state, respectively. These reactions were obtained from DFT/ $DMol^3$ with GGA-PBE functional and basis set DNP 4.4 level.

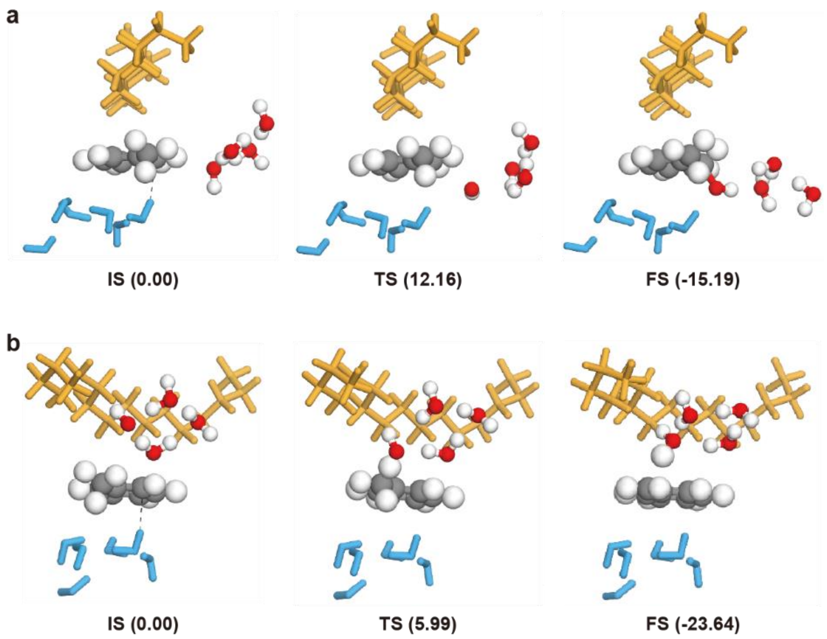

Supplementary Fig. 13. The reaction state configurations of toluene at the oil-water interfaces. **a,** OH-addition and **b,** H-abstraction. Toluene and OH· with three water molecules are shown by ball-and stick model, while the oil and water solvent molecules are described by stick model. In the color scheme, C, O, and H atoms of toluene and OH· with three water molecules are colored in gray, red and white, respectively. However, the oil and water solvent molecules are colored in orange and sky blue, respectively. The activation energy and heat of reaction are represented by the number in parenthesis below each snapshot in the unit of kcal/mol. IS, TS, and FS in each reaction mechanism represent initial state, transition state, and final state, respectively. Black dash line in the figures of IS represents the π-hydrogen interaction between the water and toluene molecules. These reactions were obtained from DFT/ DMol^3 with GGA-PBE functional and basis set DNP 4.4 level.

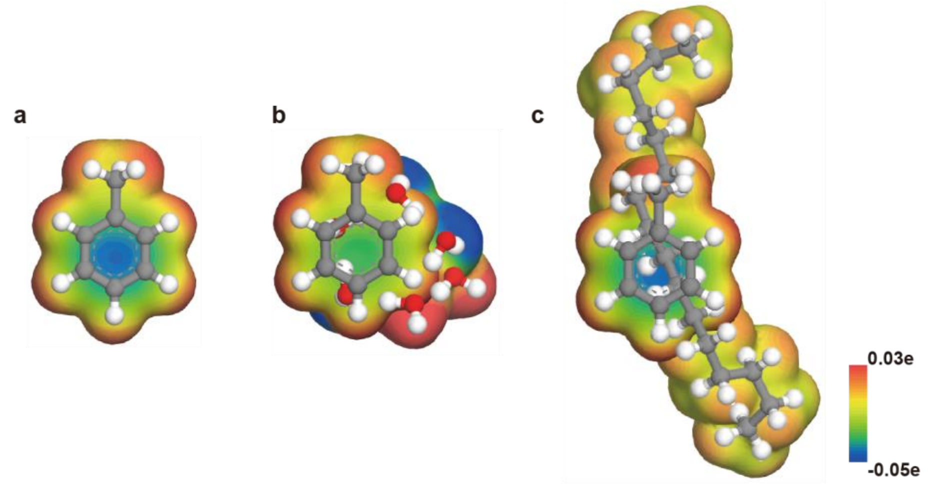

Supplementary Fig. 14. DFT optimized structure and molecular electrostatic potential (MEP) map. a, single toluene molecule. **b,** one toluene and six water molecules. **c,** one toluene and oil molecules. The MEP map provides the recognition of electron distribution in molecules. Blue color shows the negatively charged regions while red color presents the positively charged regions of MEP. For clear view, all atoms are represented by ball-and-stick model. The C, O, and H atoms are colored in gray, red, and white, respectively. These reactions were obtained from DFT/ $DMol^3$ with GGA-PBE functional and basis set DNP 4.4 level.

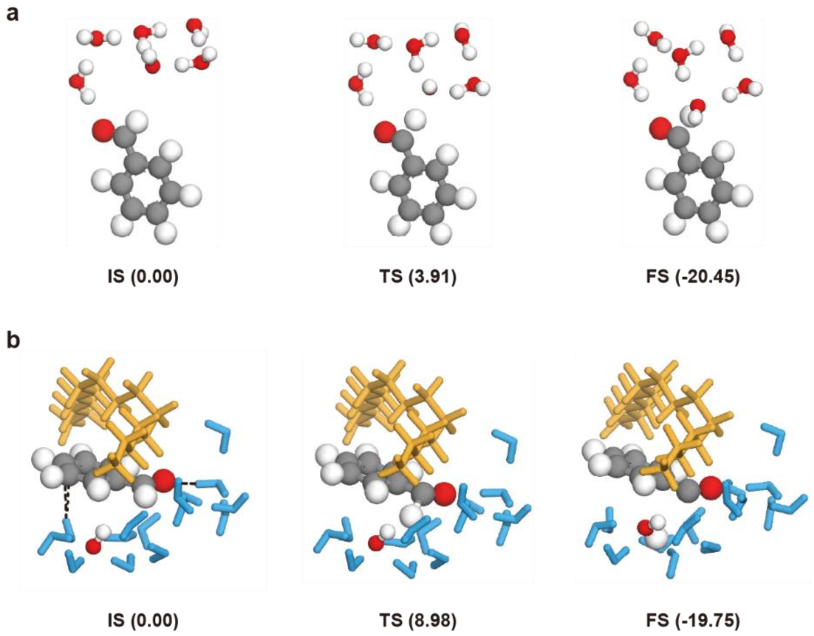

Supplementary Fig. 15. Configurations of the H-abstraction reaction states of benzaldehyde. **a,** the bulk oil and **b,** the oil-water interface. Benzaldehyde and OH· molecules are shown by ball-and stick model, and oil and water solvent molecules are described by stick model. In the color scheme, C, O, and H atoms of benzaldehyde and OH· molecules are colored in gray, red and white, respectively. However, the oil and water solvent molecules are colored in orange and sky blue, respectively. The activation energy and heat of reaction are represented by the number in parenthesis below each snapshot in the unit of kcal/mol. IS, TS, and FS in each reaction mechanism represent initial state, transition state, and final state, respectively. Black dash line in the IS figure in the oil-water interface represents the hydrogen bonding interactions between the water and benzaldehyde molecules. These reactions were obtained from DFT/ DMol^3 with GGA-PBE functional and basis set DNP 4.4 level.

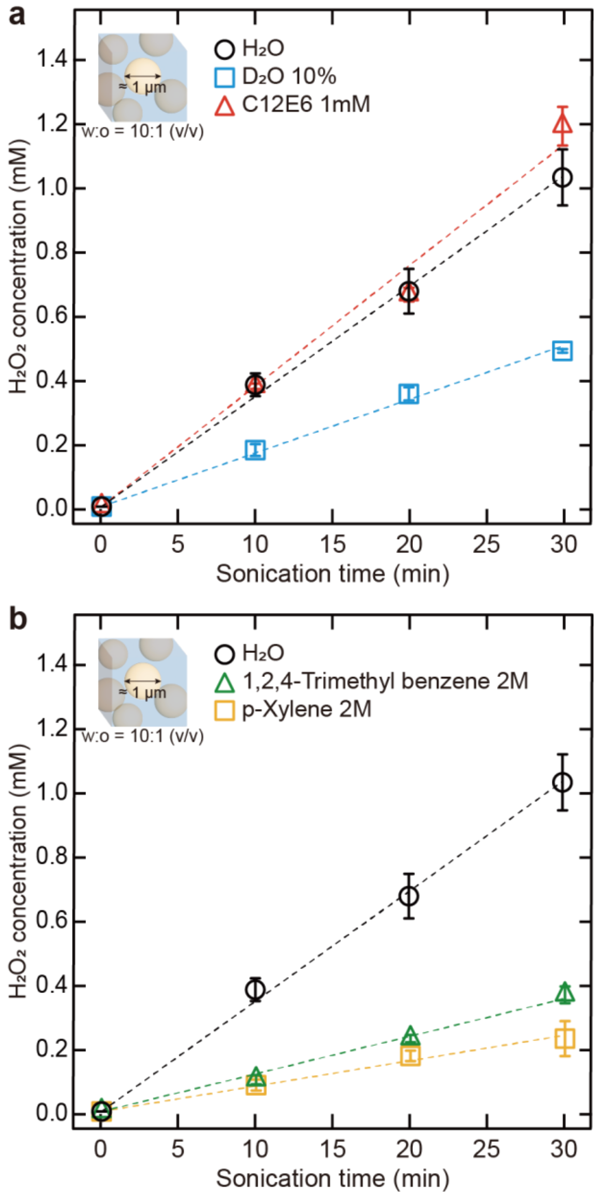

Supplementary Fig. 16. Evaluation of H_2O_2 production at various oil-water interfaces. a, b, Impact of interfacial properties (**a**) and dissolved aromatic compounds (**b**) on the formation of H_2O_2. The standard deviation of three measurements is denoted by each error bar.

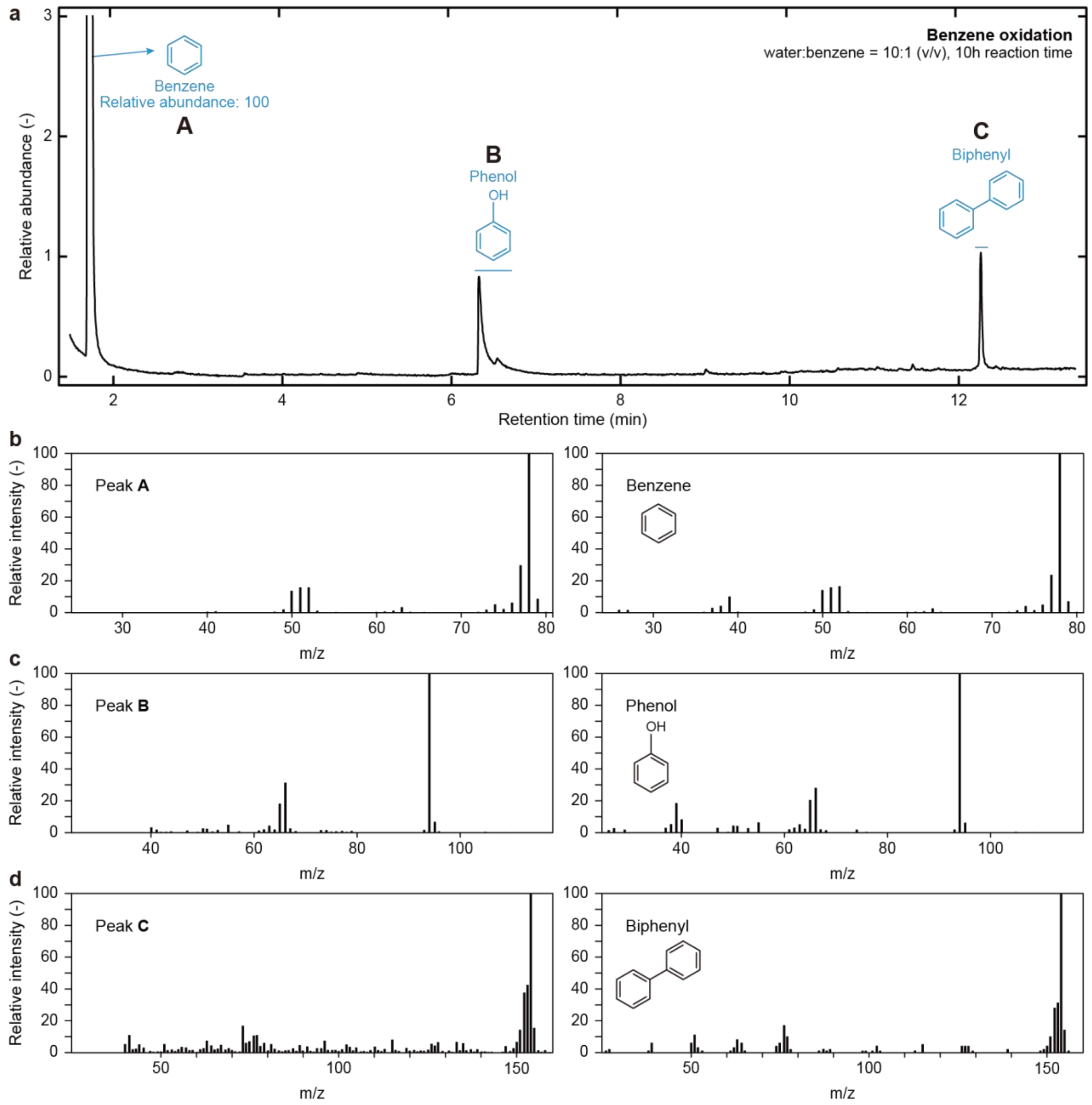

Supplementary Fig. 17. GC-MS analysis of benzene oxidation on water. a, Elution profiles of GC measurements. **b-d**, Mass spectra of the oxidized products: 1 atm oxygen, 25 ^{o}C (298 K), 10 M benzene, water to benzene ratio 10:1 (v/v), 10 h reaction.

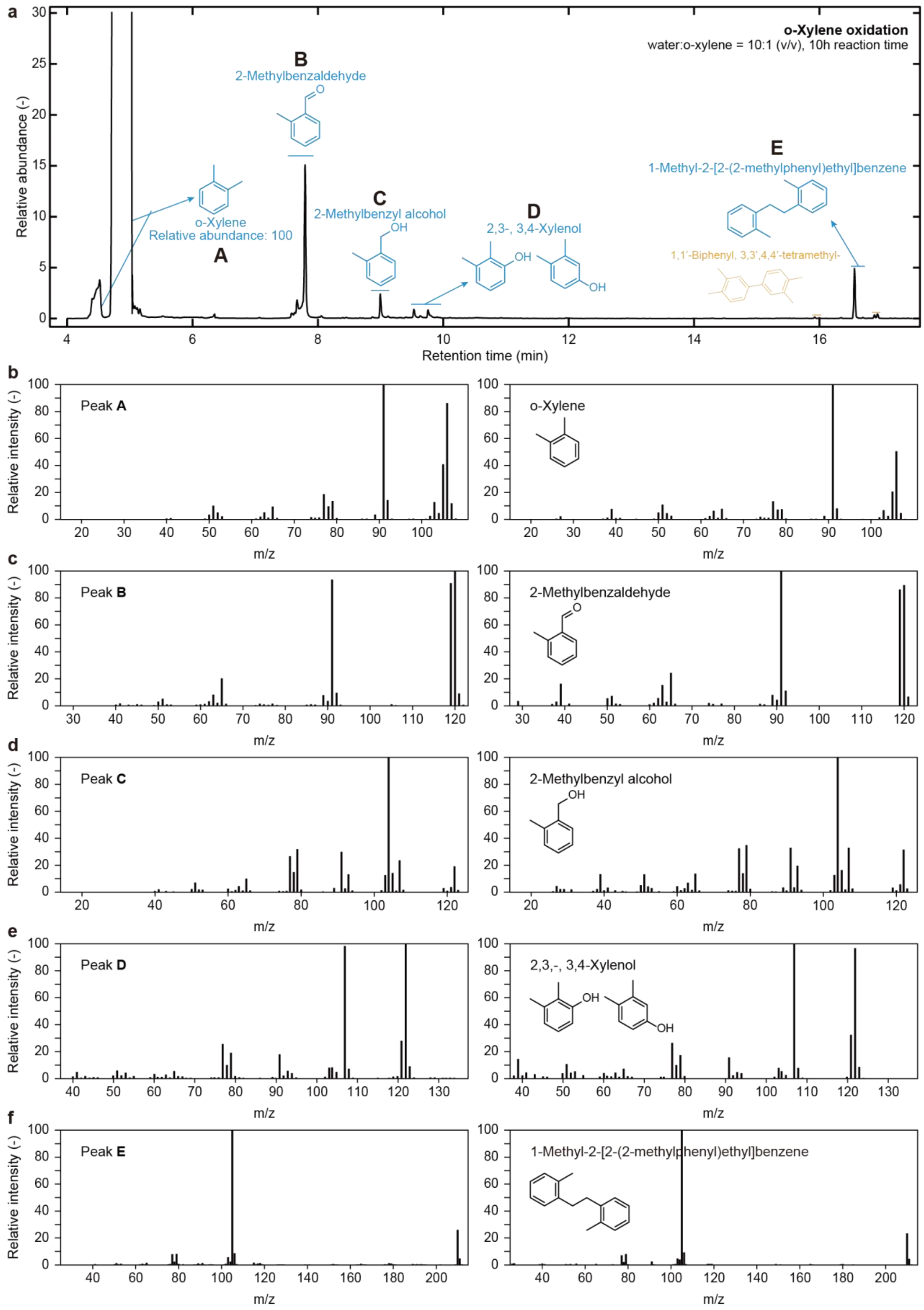

Supplementary Fig. 18. GC-MS analysis of o-xylene oxidation on water. a, Elution profiles of GC measurements. **b-f**, Mass spectra of the oxidized products: 1 atm oxygen, 25 ºC (298 K), 10 M o-xylene, water to o-xylene ratio 10:1 (v/v), 10 h reaction.

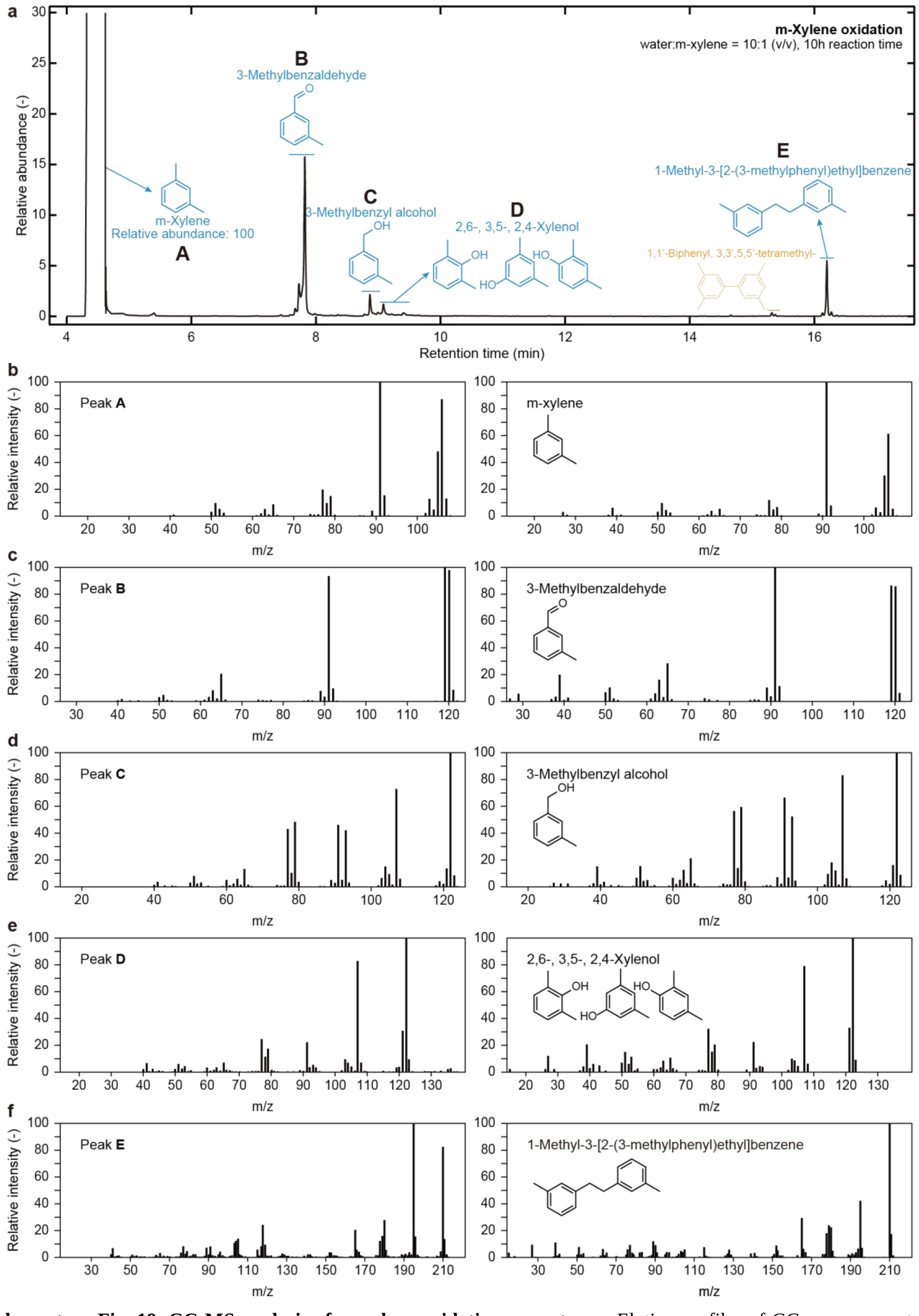

Supplementary Fig. 19. GC-MS analysis of m-xylene oxidation on water. a, Elution profiles of GC measurements. **b-f**, Mass spectra of the oxidized products: 1 atm oxygen, 25 ºC (298 K), 10 M m-xylene, water to m-xylene ratio 10:1 (v/v), 10 h reaction.

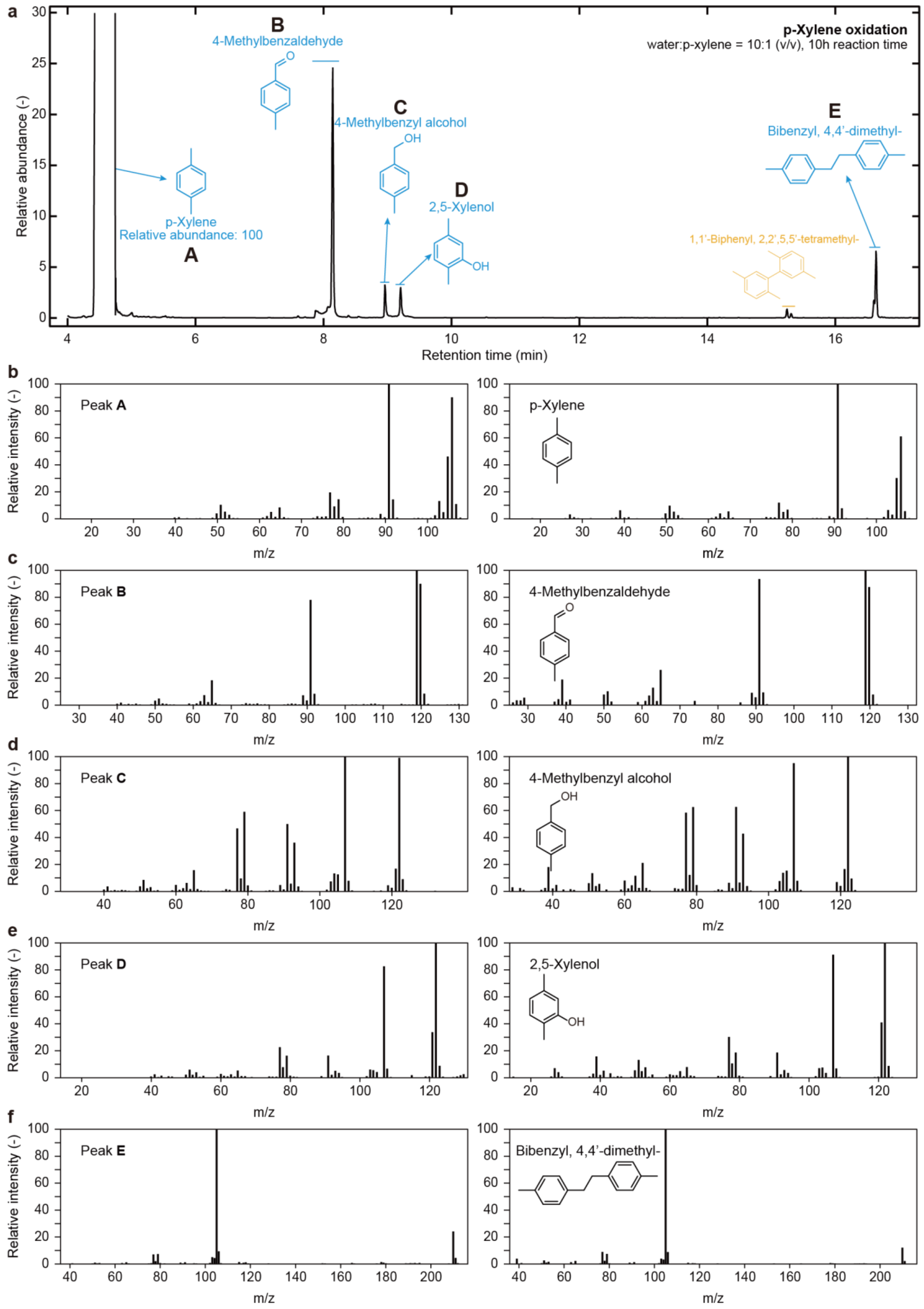

Supplementary Fig. 20. GC-MS analysis of p-xylene oxidation on water. a, Elution profiles of GC measurements. **b-f**, Mass spectra of the oxidized products: 1 atm oxygen, 25 ºC (298 K), 10 M p-xylene, water to p-xylene ratio 10:1 (v/v), 10 h reaction.

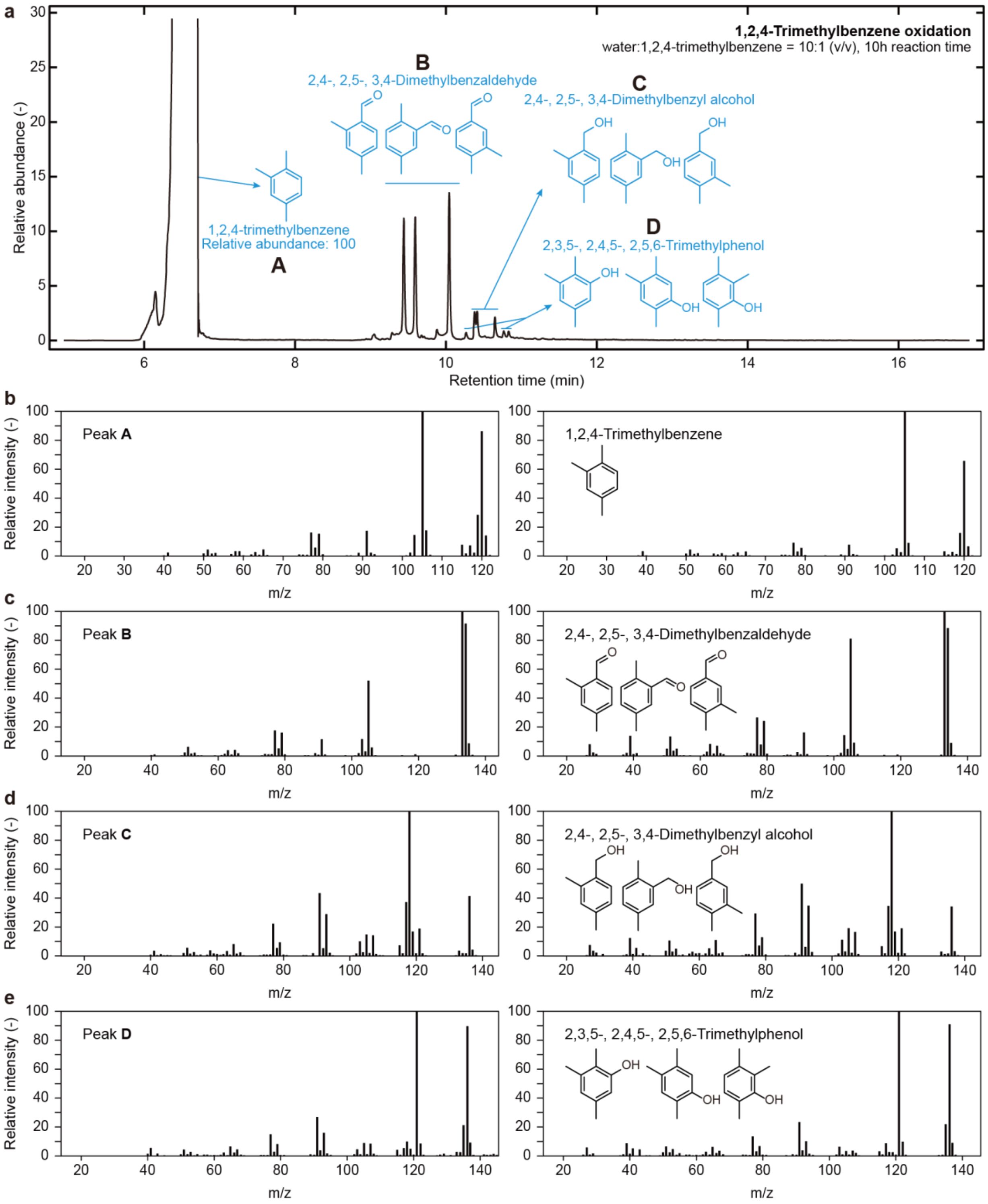

Supplementary Fig. 21. GC-MS analysis of 1,2,4-trimethylbenzene oxidation on water. a, Elution profiles of GC measurements. **b-e**, Mass spectra of the oxidized products: 1 atm oxygen, 25 ºC (298 K), 10 M 1,2,4-trimethylbenzene, water to 1,2,4-trimethylbenzene ratio 10:1 (v/v), 10 h reaction.

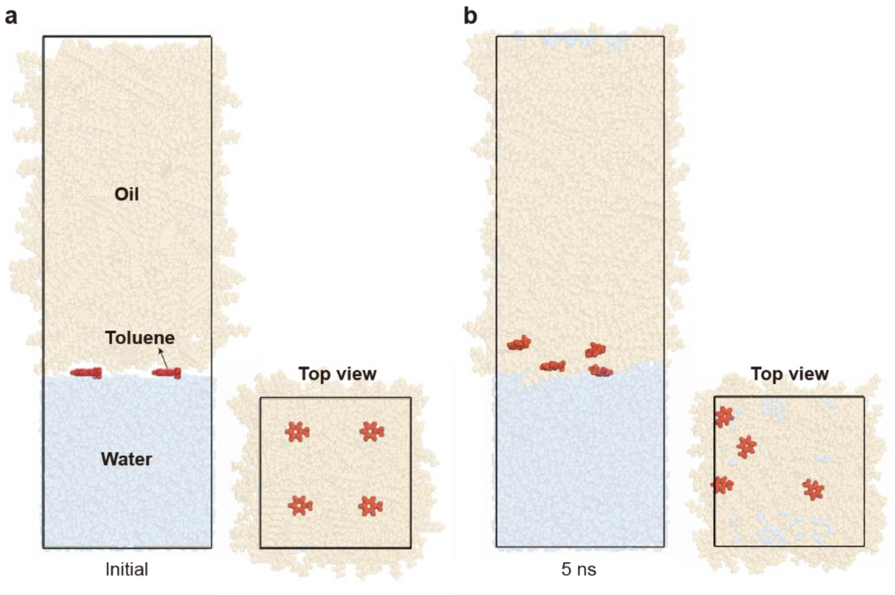

Supplementary Fig. 22. Molecular dynamics system with toluene at the oil-water interface. Snapshots of NVT-MD simulation of **a,** initial and **b,** after 5 ns with front and top views. For the clear view, the oil, toluene, and water molecules are assigned by the orange, red, and blue colored ball-and stick models, respectively.

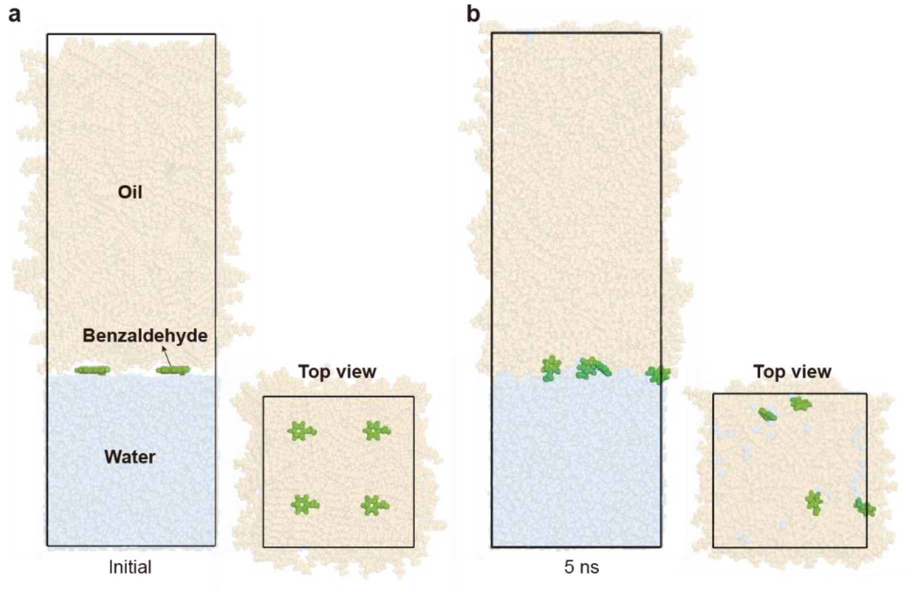

Supplementary Fig. 23. Molecular dynamics system with benzaldehyde at the oil-water interface. Snapshots of NVT-MD simulation of **a**, initial and **b**, after 5 ns with front and top views. For the clear view, the oil, benzaldehyde, and water molecules are assigned by the orange, green and blue colored ball-and stick models, respectively.

Supplementary Table 1. On water activity for selective toluene oxidation

Entry	w:o (v/v)	$[Toluene]_0$ (M)	Time (h)	Conv. (%)[a]	Selectivity (%)[a]				
					Benzaldehyde	Benzyl alcohol	Cresol	Bibenzyl	Others
1	10:1	0.01	1	4.71	> 99	-	-	-	-
2	10:1	0.01	3	13.18	> 99	-	-	-	-
3	10:1	0.01	5	22.81	> 99	-	-	-	-
4	10:1	0.01	7	30.77	> 99	-	-	-	-
5	10:1	0.01	10	48.15	> 99	-	-	-	-
6	10:1	0.01	13	61.76	> 99	-	-	-	-
7	10:1	0.01	17	86.36	> 99	-	-	-	-
8	10:1	0.01	20	> 99	> 99	-	-	-	-
9	10:1	0.01	30	> 99	> 99	-	-	-	-
10	1:1	0.01	1	1.52	> 99	-	-	-	-
11	1:1	0.01	5	10.00	> 99	-	-	-	-
12	1:1	0.01	10	23.88	> 99	-	-	-	-
13	1:10	0.01	1	0.88	> 99	-	-	-	-
14	1:10	0.01	5	2.97	> 99	-	-	-	-
15	1:10	0.01	10	4.37	> 99	-	-	-	-

All reactions were conducted under 1 atm oxygen and 25 ^{o}C (298 K). [a]Conversion and selectivity were analyzed by gas chromatography-mass spectrometry.

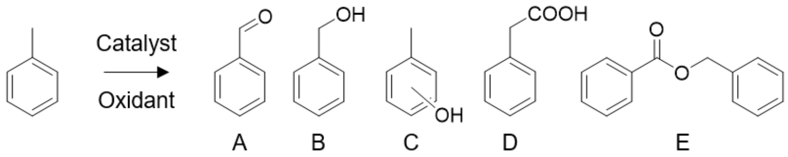

Supplementary Table 2. Comparison of on water toluene oxidation with catalytic studies

Entry	Catalyst	Oxidant	Temp. (°C)	P_{O2} (bar)	Time (h)	Yield (%)	Selectivity (%)						Ref.
							A	B	C	D	E	Others	
1	-	OH·, O_2	25	1	20	> 99	> 99	-	-	-	-	-	This work
2	Au-Pd/C	O_2	160	10	27	80	1	< 1	-	13	86	-	Ref. 1
3	Pt	O_2	160	10	5	2	20	17	-	62	1	-	Ref. 2
4	g-C_3N_4	O_2	160	10	16	2	> 99	-	-	-	-	-	Ref. 3
5[a]	*p*-BWO	O_2	25	1	6	44	81	4	-	15	-	-	Ref. 4
6[a]	TiO_2	O_2	25	1	4	< 1	25	3	-	72	-	-	Ref. 5
7[a]	Nb_2O_5-N	O_2	40	1	12	1	96	-	-	4	-	-	Ref. 6
8[b]	$[Ni(tepa)]^{2+}$	H_2O_2	60	-	24	1	10	-	90	-	-	-	Ref. 7
9[c]	$[\gamma\text{-}PW_{10}O_{38}V_2(\mu\text{-}OH)_2]^{3-}$	H_2O_2	60	-	1	1	< 1	2	86	-	-	12[d]	Ref. 8
10[b]	$MnWO_4$	H_2O_2	80	-	24	54	90	8	2	-	-	-	Ref. 9

[a]Photocactalyst. [b]Reaction was performed under atmospheric pressure. [c]Reaction was conducted under 1 atm Ar. [d]Methyl-p-benzoquinone.

Supplementary Table 3. Influence of dissolved oxygen concentration on toluene oxidation

Entry	P_{gas} (1 bar)	w:o (v/v)	$[Toluene]_0$ (M)	Time (h)	Conv. (%)[d]	Selectivity (%)[d]				
						Benzaldehyde	Benzyl alcohol	Cresol	Bibenzyl	Others
1[a]	O_2	10:1	10.00	10	2.80	24.68	2.71	4.19	25.18	43.24
2[b]	Air	10:1	10.00	10	1.74	20.76	2.90	4.36	27.00	44.98
3[c]	N_2	10:1	10.00	10	1.82	21.16	3.76	5.29	25.39	44.44
4[a]	O_2	10:1	0.01	10	48.15	> 99	-	-	-	-
5[b]	Air	10:1	0.01	10	11.90	91.74	8.26	-	-	-
6[c]	N_2	10:1	0.01	10	9.24	86.00	14.00	-	-	-

[a]Purged for 20 min with oxygen prior to emulsification. [b]Purged for 20 min with air before emulsification. [c]Purged for 20 min with nitrogen before emulsification. [d]Conversion and selectivity were analyzed by gas chromatography-mass spectrometry.

Supplementary Table 4. Effect of the π-hydrogen bond strength on toluene oxidation

Entry	Reactant	$[Reactant]_0$ (M)	Time (h)	Conv. (%)[c]	Selectivity (%)[c]				
					Aldehyde	Alcohol	Phenol	Bibenzyl	Others
1[a]	Toluene	2.00	10	6.59	44.23	0.49	0.48	32.69	22.11[d]
2[b]	Toluene	2.00	10	4.65	45.63	1.51	4.56	27.36	20.52[d]
3	Toluene	2.00	10	5.27	46.12	3.67	2.40	24.91	23.00[d]
4	p-Xylene	2.00	10	4.96	66.67	-	1.33	32.00	-
5	1,2,4-Trimethylbenzene	2.00	10	2.42	> 99	-	-	-	-

All reactions were conducted under 1 atm oxygen, 25 °C (298 K), and w:o = 10:1 (v/v). [a]D_2O 10%. [b]$C_{12}E_6$ 1mM. [c]Conversion and selectivity were analyzed by gas chromatography-mass spectrometry. [d]Other products include dimetehylbiphenyl and methyl diphenylmethane.

Supplementary Table 5. Impact of chain length of hydrocarbon oils on toluene oxidation

Entry	w:o (v/v)	$[Toluene]_0$ (M)	Time (h)	Conv. (%)[a]	Selectivity (%)[a]				
					Benzaldehyde	Benzyl alcohol	Cresol	Bibenzyl	Others
C16	10:1	0.01	10	48.15	> 99	-	-	-	-
C12	10:1	0.01	10	7.42	> 99	-	-	-	-
C8	10:1	0.01	10	0.10	> 99	-	-	-	-

All reactions were conducted under 1 atm oxygen and 25 ºC (298 K). [a]Conversion and selectivity were analyzed by gas chromatography-mass spectrometry.

Supplementary Table 6. Selective activation of C(sp^3)-H bonds in various aromatic compounds.

Entry	Reactant	[Reactant]$_0$ (M)	Time (h)	Conv. (%)[a]	Selectivity (%)[a]				
					Aldehyde	Alcohol	Phenol	Bibenzyl	Others
1	Benzene	0.01	10	-	-	-	-	-	-
2	Benzene	10.00	10	2.86	-	44.48	-	-	55.52[b]
3	o-Xylene	0.01	10	16.92	> 99	-	-	-	-
4	o-Xylene	10.00	10	3.71	55.87	8.66	6.42	25.14	3.91[c]
5	m-Xylene	0.01	10	12.50	> 99	-	-	-	-
6	m-Xylene	10.00	10	4.54	58.33	7.43	6.31	25.23	2.70[d]
7	p-Xylene	0.01	10	15.54	> 99	-	-	-	-
8	p-Xylene	10.00	10	5.31	54.67	6.29	7.05	28.95	3.05[e]
9	1,2,4-Trimethylbenzene	0.01	10	14.27	> 99	-	-	-	-
10	1,2,4-Trimethylbenzene	10.00	10	5.09	78.43	16.33	5.24	-	-

All reactions were conducted under 1 atm oxygen, 25 ^{o}C (298 K), and w:o = 10:1 (v/v). [a]Conversion and selectivity were analyzed by gas chromatography-mass spectrometry. [b]Biphenyl. [c]3,3',4,4'-Tetramethylbiphenyl. [d]3,3',5,5'-Tetramethylbiphenyl. [e]2,2',5,5'-Tetramethylbiphenyl.

Supplementary References

1. Kesavan, L. *et al.* Solvent-free oxidation of primary carbon-hydrogen bonds in toluene using Au-Pd alloy nanoparticles. *Science* **331**, 195-199 (2011).
2. Huda, M., Minamisawa, K., Tsukamoto, T., Tanabe, M. & Yamamoto, K. Aerobic toluene oxidation catalyzed by sub-nano metal particles. *Angew. Chem. Int. Ed.* **58**, 1002-1006 (2019).
3. Li, X.-H., Wang X. & Antonietti M. Solvent-free and metal-free oxidation of toluene using O_2 and g-C_3N_4 with nanopores: nanostructure boosts the catalytic selectivity. *ACS Catal.* **2**, 2082-2086 (2012).
4. Cao, X. *et al.* A photochromic composite with enhanced carrier separation for the photocatalytic activation of benzylic C–H bonds in toluene. *Nat. Catal.* **1**, 704-710 (2018).
5. Tripathy, J., Lee, K. & Schmuki, P. Tuning the selectivity of photocatalytic synthetic reactions using modified TiO_2 nanotubes. *Angew. Chem. Int. Ed.* **53**, 12605-12608 (2014).
6. Su, K. *et al.* Visible-light-driven selective oxidation of toluene into benzaldehyde over nitrogen-modified Nb_2O_5 nanomeshes. *ACS Catal.* **10**, 1324-1333 (2020).
7. Morimoto, Y., Bunno, S., Fujieda, N., Sugimoto, H. & Itoh, S. Direct hydroxylation of benzene to phenol using hydrogen peroxide catalyzed by nickel complexes supported by pyridylalkylamine ligands. *J. Am. Chem. Soc.* **137**, 5867–5870 (2015).
8. Kamata, K., Yamaura, T. & Mizuno, N. Chemo- and regioselective direct hydroxylation of arenes with hydrogen peroxide catalyzed by a divanadium-substituted phosphotungstate. *Angew. Chem. Int. Ed.* **51**, 7275-7278 (2012).
9. Mal, D. D., Khilari, S. & Pradhan, D. Efficient and selective oxidation of toluene to benzaldehyde on manganese tungstate nanobars: a noble metal-free approach. *Green Chem.* **20**, 2279-2289 (2018).
